# The effect of air pollution on convenience-based or other-oriented lies

**DOI:** 10.1371/journal.pone.0216238

**Published:** 2019-04-29

**Authors:** Song Wu, Tingbin Wang

**Affiliations:** 1 College of Psychology and Sociology, Shenzhen University, Shenzhen, China; 2 School of Psychology, University of Birmingham, Birmingham, United Kingdom; Middlesex University, UNITED KINGDOM

## Abstract

Besides endangering human health, air pollution has profound effects on individuals’ cognition, emotions, and behavior. Previous studies have found that air pollution could increase self-oriented lies. We summarized two explanations for this phenomenon: (1) air pollution makes people less likely to regard lies as unethical, and (2) air pollution makes people more likely to approach materials rewards. The present study mainly measured three kinds of lies—self-convenience, other-convenience, and other-material lies—to investigate these two explanations. Participants were asked to imagine living in either a polluted or a clean situation in two online studies and one laboratory study. The results showed that air pollution did not influence self-convenience lies (Studies 1 and 2), and clean air increased both other-convenience and other-material lies (Studies 2 and 3). According to these results, both explanations are supported. The theoretical implications of the present study are discussed.

## Introduction

Air pollution is a serious global problem. Besides the health and environmental concerns caused by air pollution, psychologists are also interested in how air pollution can influence individuals’ social cognitions and behaviors. Air pollution has been shown to promote unethical behaviors [1–4], including as lying [5]. Specifically, Lu, Lee (5) found that individuals were more likely to lie in order to receive a monetary reward when imagining living in a scenario with high level of air pollution than they would in a clean-air scenario. The present study aimed to extend these earlier studies by examining whether air pollution could increase specifically convenience-based and other-oriented lies.

### Theory framework

From previous studies, which mainly measured self-interest lies that are unethical but profitable, we distill two possible routes through which air pollution increases lying behaviors: (1) air pollution makes people less likely to regard lies as unethical, and (2) air pollution makes people more likely to approach material rewards. We argued that both routes, discussed in more detail below, could reliably explain the negative impact of air pollution on ethical behavior.

Firstly, air pollution may decrease individuals’ consideration for moral and social rules and lead them to believe that rule transgressing is common, acceptable, or undetectable (*rule-route*). The broken window theory suggests that rule-breaking behaviors implied in disorderly environments can spread to other domains and promote more rule-breaking behaviors [6–8]. It has also been proposed that social reasoning (e.g., the argument that rule-transgressing is common and acceptable in a given community) plays an important role in the way in which disorder spreads [7, 9]. In addition, the low visibility in air pollution can create a sense of anonymity that leads individuals to believe that misconducts are undetectable and not punished. In fact, a previous study found that individuals are more likely to cheat in a dim room than in a bright room [10]. Therefore, air pollution as a disorderly environment can also increase lying behaviors through decreasing the power of moral rules.

Secondly, air pollution may increase individuals’ consideration of and desire for behavioral outcomes, especially material ones, and lead them to transgress rules to reach such outcomes (*outcome-route*). Specifically, air pollution can increase individuals’ anxiety [11], which in turn promotes unethical behaviors [12]. In this explanation, anxiety suggests a potential threat to the self, and acquiring more material resources (e.g., money) would be a means to cope with the anxiety and potential threat [12]. Therefore, air pollution can promote lying behaviors that generate more resources. More importantly, previous studies have found that anxiety mediated the effect of air pollution on lying behaviors [5].

Although both theoretical explanations and empirical results suggest that air pollution increase lying behaviors, it is still unclear whether the effect of air pollution is valid regarding all kinds of lies. A simple logic is that if the effect of air pollution disappears when a route is obstructed, then this route provides a reliable explanation. However, since previous studies mostly relied on scenarios where lies held financial benefit for individuals (self-material lies), the results could not examine either rule- or outcome-routes because neither route was hindered. In order to extend previous findings on the effect of air pollution on ethical behavior, we modified the measurement of lying behaviors by (1) decreasing the attractiveness of the material reward attached to lying.

### Types of lying

We employed other-oriented lies to position the lying behaviors as less unethical. For the purposes of this study, other-oriented lies were defined as altruistic white lies that work in favor of the other, at the cost of the liar [13]. Other-oriented lies involve a moral dilemma in that the liar is compelled to use immoral methods to attain moral outcomes. Previous studies found that other-oriented lies are perceived to be a relatively moral behavior [14], positively correlated with altruism[13], and even more morally justified than a selfish truth [15]. Besides, other-oriented lies, as prosocial behaviors, may improve the liar’s reputation, foster trust, and bring future reciprocal rewards [15, 16]. Some studies even found that individuals are more likely to lie for the benefit of others than their own [17, 18]. These results suggest that other-oriented lying is a moral-conforming behavior and regarded as less unethical than self-oriented lying, and it could be used to examine the rule-route described earlier. We argued that changing the beneficiary of the lying behavior from self to others would hinder the rule-route of the air pollution effect.

On the other hand, convenience-based lies were used to reduce the attractiveness of the rewards for lying. Convenience-based lies are those that could save time and energy but do not generate any material rewards. For example, if completing a simple task and a complex task are rewarded with the same amount of money, a person may tell a lie to perform the simple task instead of the complex one. Because convenience-based lies do not usually bring obvious material rewards, its outcomes do not serve as effective resources for coping with the anxiety caused by air pollution. A previous study found that people were reluctant to lie just to attain convenience, even when they were in state of ego depletion [19]. In addition, research participants were more likely to employ material-based lies than convenience-based lies [19]. These results suggest that convenience is less attractive than material reward, and thus, changing the outcome from a monetary reward to increased convenience would obstruct the outcome-route of the air pollution effect.

Considering the outcome and beneficiary of lying behaviors, we can distinguish between four kinds of lies: self-material, self-convenience, other-material, and other-convenience. Since self-material lies cannot provide substantial evidence for either proposed rule, the focus of the present study is on the remaining three types of lies. We proposed that air pollution could not increase self-convenience lies because the outcome-route would be hindered. In contrast, it was proposed that air pollution may decrease other-material and other-convenience lies. Three reasons can account for this reversed effect. Firstly, individuals in an air-pollution condition may be more likely to transgress the rule of helping others because air pollution implies that rule breaking is common and acceptable. Secondly, the sense of anonymity caused by air pollution may decrease prosocial behaviors because of the reduced possibility of being identified and reciprocated. Thirdly, if the lying outcomes are material resources, in order to cope with anxiety caused by air pollution, individuals may be less likely to help others in order to avoid increasing relative disparity.

### Overview

The present study aimed to examine the effect air pollution on convenience-based and other-oriented lies. It was hypothesized that (1) there is no effect of air pollution on self-convenience lies; (2) individuals in an air-pollution condition will be less likely to tell lies to help others obtain convenience gains (other-convenience lies), compared to those in a clean-air condition; and (3) individuals in an air-pollution condition will be less likely to tell lies to help others gain money (other-material lies), compared to those in a clean-air condition.

## Study 1

In Study 1, participants’ experiences of either clean or polluted air were manipulated to examine whether air pollution could increase the use of self-convenience lies.

### Method

#### Participants and design

Participants were 182 adults (107 females; average age = 32.55 ± 6.98 years), recruited from a professional data collection company (https://www.wjx.cn/). They received remuneration from the company for their participation. A one-factor (air quality: pollution vs. clean) between-subjects design was employed. Since previous results found the effect size (Cohen’s *d*) of air pollution to be between 0.29 and 0.60 [5], we set 0.45 as the effect size to calculate sample size. The sample exceeded the recommended size (*n* = 144) at .85 power (*α* = .05), calculated using the G*Power software package.

#### Procedure

All participants received a link from the data collection company to take part in the study. Participants first reported their demographic information, and then were randomly assigned to either the clean-air or the air-pollution condition. The manipulation of air quality has been used in previous studies [5].

Participants in the air-pollution condition were presented with nine photos of Beijing featuring a polluted day, while participants in the clean-air condition were presented with nine photos of Beijing featuring a clean day. Each photo in the air-pollution condition was paired with a certain photo in the clean-air condition because each photo pair was taken at the same location. The photos were presented one by one, and participants had to rate the extent of air pollution on each photo on a 7-point scale (1 = *no pollution at all*, 7 = *very much polluted;* Cronbach’s *α* was 0.96 in Study 1, 0.99 in Study 2, and 0.96 in Study 3). After this, participants were asked to write a fictional diary entry about living in this city with the air-quality condition depicted on the photos. All nine photos were presented again when participants were writing their diaries. This diary task was used before and proved to be valid [5].

After the manipulation, participants had to select their birth month among six options (1 = *January and February*, 2 = *March and April*, 3 = *May and June*, 4 = *July and August*, 5 = *September and October*, 6 = *November and December*). Participants were told that they would be assigned to one of six groups according to their answer, and different groups would have to answer different questions. Importantly, participants were informed that the bigger the number they selected, the more questions they would have to complete. The task ended immediately after participants answered this question and pressed the “next page” button.

A pilot survey (*n* = 285, 140 females; average age = 32.89 ± 7.73 years) was conducted through the same data collection company to determine the base level of birth month question of this company sample. Participants were asked to report their birth month honestly. We coded the answers (1 = *January and February*, 2 = *March and April*, 3 = *May and June*, 4 = *July and August*, 5 = *September and October*, 6 = *November and December*), and calculated the mean and standard deviation of those coded scores to identify the base level of birth month (*M* = 3.65, *SD* = 1.67, 95% CI = [3.45, 3.84]). We also analyzed the census data provided by National Bureau of Statistics of China [20, 21]. The data contains the number of populations born in each month in China from November 1, 1999 to October 31, 2000 and from November 1, 2009 to October 31, 2010. According to the coding and calculational methods mentioned above, the base level of birth month is 3.67 which is very close to the result of our pilot study. Considering the sample source and participants’ ages, we decided to use the result of pilot study as a base level in Studies 1 and 2.

#### Ethical considerations

All three studies and the pilot study were approved by the institutional review board of the first author’s university and form part of a larger project. Since the pilot study and Studies 1 and 2 were completed online, participants indicated their consent by clicking on the “confirm” button after they were informed that this was a scientific study, the risks and benefits of participating in it, and that they could withdraw at any time without repercussions. We obtained verbal consent from participants in Study 3 as this was necessary to maintain anonymity. The research team recorded participants’ verbal consent.

### Results

Nine participants failed to complete the diary writing task appropriately and their data were excluded from the analyses. Specifically, three participants used nonsensical letters (e.g., fjoiejajoef), two participants refused to write (for example, one participant wrote “it is difficult to imagine”), and four participants assigned to the clean-air condition wrote a diary entry about living in a polluted environment.

#### Manipulation check

An independent-samples *t* test revealed that participants evaluated the air quality in the air-pollution condition (*M* = 5.34, *SD* = 0.70) to be significantly more polluted than that in the clean-air condition (*M* = 2.54, *SD* = 0.83), *t*(171) = 23.91, *p* < .001, *d* = 3.65, 95% CI = [2.57, 3.03]. This result suggests that our manipulation was valid.

#### Self-reported birth month

An independent-samples *t* test was conducted to examine the effect of air pollution (clean air vs. air pollution) on lying behaviors. The scores of self-reported birth month in the air-pollution condition (*M* = 3.62, *SD* = 1.76, 95% CI = [3.25, 4.00]) were not significantly different from those in the clean-air condition (*M* = 3. 33, *SD* = 1.78, 95% CI = [2.94, 3.71]), *t*(171) = 1.10, *p* = .274, *d* = 0.16. In addition, the scores of self-reported birth month in both conditions were not significantly different from the base level (*M* = 3.65, *SD* = 1.67)—air-pollution condition: *t*(370) = 0.14, *p* = .891, *d* = 0.02; clean-air condition: *t*(369) = 1.55, *p* = .122, *d* = 0.19.

#### Gender differences

In order to analyze the gender differences, a 2 (air quality: air pollution vs. clean air) × 2 (gender: male vs. female) analysis of variance was conducted with self-reported birth month as the dependent variable. The main effect of gender was found not to be significant, *F*(1,169) = 0.74, *p* = .390, $\eta_{p}^{2}$ < 0.01, and the interaction effect of gender and air quality was not significant either, *F*(1,169) = 0.08, *p* = .779, $\eta_{p}^{2}$ < 0.01.

### Discussion

These results supported our first hypothesis, as air quality did not affect self-convenience lies. This may be because the convenience outcome (answering fewer questions) was not as attractive and valuable as a material outcome (more money) and was harder to be used as a means of coping with anxiety and threats.

In Study 2, we further examined whether air quality influenced both self- and other- convenience lies. One might argue that the photos of Beijing were inappropriate for Chinese participants, as participants were very familiar with the city and may have had preconceived ideas about it. In order to exclude this possibility, we also employed a measurement of lying behaviors used in previous studies to evaluate our manipulation.

## Study 2

Study 2 aimed to replicate and extend the results of Study 1 by examining both self- and other-convenience lies. Moreover, we included a questionnaire about lying behavior in Study 2 to ensure that our manipulation was valid. This questionnaire was used in a previous study [5]; thus our results could be compared with previous ones.

### Method

#### Participants and design

Participants were 165 adults (76 females; average age = 34.58 ± 8.38 years), recruited from a professional data collection company (https://www.wjx.cn/). They received remuneration from the company for their participation. A 2 (air quality: air pollution vs. clean air) × 2 (lying style: self-oriented vs. other-oriented) between-subjects design was employed. As in Study 1, we set effect size *f* at 0.25 to calculate the sample size. The sample exceeded the recommended size (*n* = 146) at .85 power (*α* = .05) according to G*Power.

#### Procedure

The manipulation of air quality and measurement of lying behaviors were the same as in Study 1. Participants were assigned to either the self-oriented or the other-oriented condition. Participants in the self-oriented condition were told that their choices would determine their own number of questions. On the other hand, participants in the other-oriented condition were told that their number of questions was determined by a prior participant and their choices would also determine the next participant’s number of questions. Subsequently, all participants completed a questionnaire to assess the tendency to lie, which included 16 items from the Self-Reported Inappropriate Negotiation Strategies (SINS) scale. These 16 items are organized in four dimensions (false promises, misrepresentation, strategic misrepresentation of positive emotion, strategic misrepresentation of negative emotion) in the SINS. This scale is regarded as a measurement of self-material lies and its reliability has been proved previously [5, 22]. Participants were asked to imagine that they were engaging in a negotiation that was very important to them and their business. They had to indicate the extent to which they thought that each tactic was appropriate to be used by them in such situation on a 7-point scale (1 = *not at all appropriate*, 7 = *very appropriate*; Cronbach’s *α* = .96).

### Results

Twelve participants did not complete the diary writing task appropriately and their data were excluded from the analyses. Specifically, five participants used nonsensical letters or characters, six participants refused to write, and one participant assigned to the clean-air condition wrote an irrelevant sentence (i.e., In the place where I work, more and more people get sick).

#### Manipulation check

An independent-samples *t* test revealed that participants evaluated the air quality in the air-pollution condition (*M* = 5.83, *SD* = 0.60) to be significantly more polluted than that in the clean-air condition (*M* = 2.02, *SD* = 0.57), *t*(151) = 40.12, *p* < .001, *d* = 6.51, 95% CI = [3.62, 3.99]. This result suggests that our manipulation was valid.

#### Self-reported birth month

A 2 (air quality: air pollution vs. clean air) × 2 (lying style: self-oriented vs. other-oriented) analysis of variance was conducted with self-reported birth month as the dependent variable. The results showed a marginally significant main effect of air quality, *F*(1,149) = 3.07, *p* = .082, $\eta_{p}^{2}$ = 0.02, a non-significant main effect of lying style, *F*(1,149) = 1.14, *p* = .288, $\eta_{p}^{2}$ < 0.01, and a marginally significant interaction effect, *F*(1,149) = 3.41, *p* = .067, $\eta_{p}^{2}$ = 0.22.

**Fig 1:**
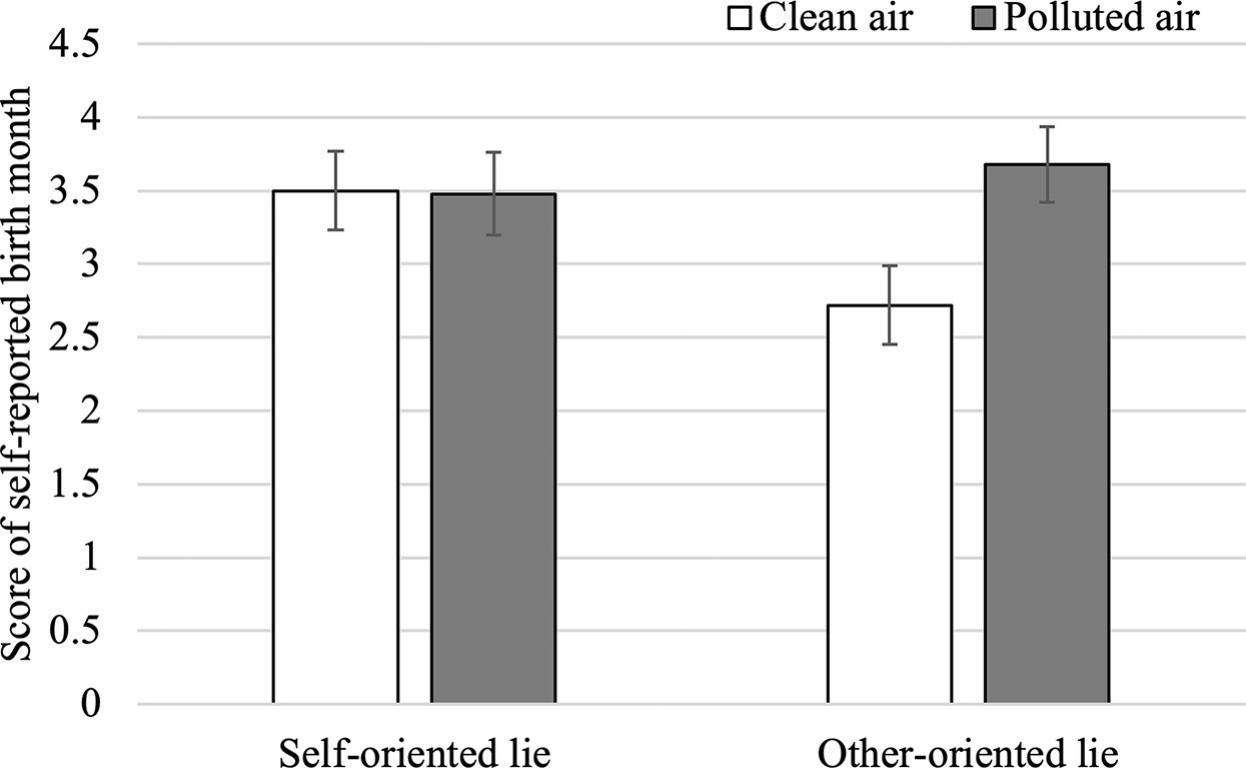
Interaction effect of air quality and lying style on the score of self-reported birth month. The error bars in the figure represent standard errors of the mean.

We further analyzed the marginally significant interaction effect because its effect size was medium to large (Fig 1). Specifically, in the self-oriented lying situation, there was no significant difference between participants in the clean-air condition (*M* = 3.50, *SD* = 1.59, 95% CI = [2.96, 4.04]) and air pollution condition (*M* = 3.48, *SD* = 1.75, 95% CI = [2.91, 4.04]) in terms of scores of self-reported birth month, *t*(74) = 0.07, *p* = .949, *d* = 0.01. However, in the other-oriented lying situation, the score of birth month in the clean-air condition (*M* = 2.72, *SD* = 1.60, 95% CI = [2.18, 3.26]) was significantly lower than that in the air-pollution condition (*M* = 3.68, *SD* = 1.63, 95% CI = [3.17, 4.20]), *t*(75) = 2.60, *p* = .011, *d* = 0.54.

We also compared self-reported birth month in each of the four conditions with the base level (*M* = 3.65, *SD* = 1.67) from the pilot study. The results revealed that only participants in the clean-air and other-oriented lie condition had a significantly lower birth month score than the base level, *t*(319) = 3.15, *p* = .002, *d* = 0.57, and the scores in all other conditions were not significant different from the base level—air-pollution and other-oriented lie: *t*(324) = 0.12, *p* = .904, *d* = 0.02; clean-air and self-oriented lie: *t*(319) = 0.51, *p* = .613, *d* = 0.09; and air-pollution and self-oriented lie: *t*(323) = 0.61, *p* = .540, *d* = 0.10.

#### Gender differences

A 2 (air quality: air pollution vs. clean air) × 2 (lying style: self-oriented vs. other-oriented) × 2 (gender: male vs. female) analysis of variance was conducted with self-reported birth month as the dependent variable. Results showed that all effects related to gender were not significant—main effect of gender: *F*(1,145) = 0.85, *p* = .357, $\eta_{p}^{2}$ < 0.01; interaction effect of gender and air quality: *F*(1,145) = 0.27, *p* = .605, $\eta_{p}^{2}$ < 0.01; interaction effect of gender and lying style: *F*(1,145) = 0.57, *p* = .451, $\eta_{p}^{2}$ < 0.01; and three-way interaction effect: *F*(1,145) = 1.87, *p* = .174, $\eta_{p}^{2}$ = 0.01.

#### Self-Reported Inappropriate Negotiation Strategies (SINS)

An independent-samples *t* test revealed that participants in the air pollution condition (*M* = 3.93, *SD* = 1.30, 95% CI = [3.64, 4.22]) had a significantly higher SINS score than did those in the air clean condition (*M* = 3.20, *SD* = 1.25, 95% CI = [2.90, 3.49]), *t*(151) = 3.53, *p* = .001, *d* = 0.57. In addition, the gender differences (*M*_male_ = 3.71, *SD*_male_ = 1.32; *M*_female_ = 3.44, *SD*_female_ = 1.33) on SINS was not significant, *t*(151) = 1.27, *p* = .205, *d* = 0.20.

### Discussion

Study 2 replicated the results of Study 1, showing that air quality did not affect self-convenience lies. However, it was found that individuals in the air-pollution condition were less likely to tell other-convenience lies than were those in the clean-air condition. Although this result seems to be a reversal of the previous effect of air pollution on lies, it has the same underlying mechanism. A detailed discussion of this is provided in the general discussion section. More importantly, consistent with previous studies [5], we found that participants in the air-pollution condition had a higher SINS score, which suggests that air pollution increased participants’ tendency to tell self-material lies. Taken together, these results demonstrate that polluted air increases self-materials lies, whereas clean air increases other-convenience lies. Therefore, we need to examine whether clean air increases other-material lies in Study 3.

## Study 3

Study 3 aimed to extend Study 2 by examining whether clean air increases other-material lies. In Study 3, participants were asked to determine the next participant's bonus by rolling a dice, and they had the opportunity to misreport the dice result to help the next participant to get more money. In order to increase the internal validity, a laboratory study was conducted.

### Method

#### Participants and design

A total of 75 students (54 females; average age = 19.01 ± 1.43 years) participated in the present study for financial remuneration. A one-factor (air quality: air pollution vs. clean air) between-subjects design was employed.

#### Procedure

Upon arrival at the laboratory, participants were asked to sit at a table, and all instructions and manipulation were presented on a computer screen. The manipulation of air quality was the same as in Studies 1 and 2. After the manipulation, participants were told that in order to encourage students to participate in psychological experiments, they were provided with an opportunity to get extra money, in addition to the 10 *yuan* (Chinese currency) for showing up. However, considering equity, each participant’s extra reward was determined by the prior one (except for the first participant’s extra reward that was determined by the experimenter). All participants were told to roll a dice in an opaque cup with a lid, and write the dice result on a sheet. The lid could not be opened, and the dice result could be identified only through a hole in the lid. Participants were told that the next participant’s extra reward was determined by multiplying the dice result by 3 yuan (i.e., 3 *yuan* for 1, 6 *yuan* for 2, … 18 *yuan* for 6). Participants did not know their own extra reward until they handed in their sheets. In fact, all participants received 9 *yuan* as the extra reward.

### Results

#### Manipulation check

An independent-samples *t* test revealed that participants evaluated the air quality in the air-pollution condition (*M* = 5.30, *SD* = 0.84) to be significantly more polluted than that in the clean-air condition (*M* = 2.90, *SD* = 0.99), *t*(70) = 12.02, *p* < .001, *d* = 2.88, 95% CI = [2.17, 3.03]. This result suggests that our manipulation was valid.

#### Lying behaviors

In order to examine the effect of air quality on other-oriented lies, an independent-samples *t* test (one-tailed) was conducted. The dice results reported by participants in the clean-air condition (*M* = 4.25, *SD* = 1.65, 95% CI = [3.69, 4.81]) were significantly higher than those in the air-pollution condition (*M* = 3.49, *SD* = 1.85, 95% CI = [2.89, 4.09]), *t*(73) = 1.88, *p* = .032, *d* = 0.44. In addition, the dice results reported by participants in the clean-air condition were significantly higher than the expected value of 3.5, *t*(35) = 2.74, *p* = .010, *d* = 0.64, while this was not the case in the air-pollution condition, *t*(38) = -0.04, *p* = .966, *d* < 0.01.

#### Gender differences

A 2 (air quality: air pollution vs. clean air) × 2 (gender: male vs. female) analysis of variance was conducted to examine the gender differences. The main effect of gender was significant: female (*M* = 4.17, *SD* = 1.72) are more likely to tell other-material lies than male (*M* = 3.05, *SD* = 1.72), *F*(1,71) = 8.59, *p* = .005, $\eta_{p}^{2}$ = 0.11. However, the interaction effect of gender and air quality was not significant, *F*(1,71) = 1.44, *p* = .235, $\eta_{p}^{2}$ = 0.02.

### Discussion

Study 3 yielded a result consistent with Study 2, showing that clean air increased other-oriented lies. Moreover, Study 3 extended the results of Study 2 from convenience-based lies to material-based lies. In addition, since Studies 1 and 2 were online studies, Study 3 also increased the internal validity of the whole research by using a well-controlled laboratory study. Considering all three studies, it is appropriate to conclude that air pollution does not always promote all kinds of lying behaviors, as it sometimes inhibits certain kinds of lies (e.g., other-oriented lies).

## General discussion

All study hypotheses were supported by the results that air pollution did not affect self-convenience lies and clean air increased other-oriented lies (both convenience and material). Although these results seemed to be inconsistent with previous results showing that air pollution increased lying behaviors [5], they actually support the same explanation of the underlying mechanisms. As mentioned before, air pollution can influence lying behaviors through two mechanisms—decreasing the effect of moral rules and increasing the desire for resources. Thus, it is reasonable to speculate that air pollution leads individuals to lie in order to gain more material rewards for themselves. However, if the lying outcome is changed into convenience rather than material rewards, air pollution should have a smaller impact, or even no impact, on self-convenience lies because convenience is less attractive and effective than materials for coping with the anxiety caused by air pollution [12, 19]. The non-significant effect of air pollution on self-convenience lies in Studies 1 and 2 supports this speculation and outcome route. Moreover, other explanations, such as culture differences, can be excluded, because the Study 2 found that polluted air increased self-material lies (the SINS) which was exact the same result as in a previous study [5].

On the other hand, changing the beneficiary of lies into others can also influence the effect of air pollution on dishonesty. It has been found that other-oriented lies were acceptable in terms of moral evaluation [14, 15], produced smaller moral conflicts [23], and happened more often than self-oriented lies in laboratory studies [17]. These results suggest that the helping component in other-oriented lies is required by moral and social rules; therefore, according to the rule-route, air pollution could inhibit other-oriented lies. In addition, as for the outcome-route, helping others to gain resources can decrease individuals’ own relative amount of resources; therefore, air pollution should also inhibit other-oriented lies through the outcome-route. The results of Study 2 and 3 support this speculation and rule-route by showing that clean air increases other-oriented lies.

Combining the present study with previous ones, it is reasonable to speculate that air pollution may lead individuals to be more competitive and have more desire for high status or advantage position. Air pollution can be seen as a threat to individuals’ health and survival, and the anxiety arising in such threat situations can push individuals to do things to cope with the threat and increase their chances to survive [11, 12]. From an evolutionary perspective, individuals with certain advantages, such as higher status and more resources, are more likely to survive than disadvantaged individuals [24]. Besides absolute advantages, relative advantages are also important. In order to achieve such advantages, individuals will become more selfish and competitive in air-pollution conditions. Therefore, as confirmed by research, air pollution promotes self-oriented lies but inhibits other-oriented lies. This could also explain why previous studies found that air quality negatively predicted violent crime [1] and aggression [2], and positively predicted trust [25]. Naturally, more studies are required to investigate these explanations in the future.

The gender differences in the results of our study also need to be considered. Previous studies have found that males are more likely to tell both self-oriented and altruistic white lies than females [13, 26]. However, the present study showed different results. Studies 1 and 2 found no gender differences on convenience lies (both self-oriented and other-oriented). This may be accounted for by a floor effect in that people did not lie for convenience. However, Study 3 found that females were more likely to tell other-material lies than males. This may be accounted by the cover story in which participants were told that their extra rewards were determined by others. It may be that females are more likely to be reciprocally altruistic than males. Gender differences related to deception should be examined further in future.
